# Caterpillars and Host Plant Records for 59 Species of Geometridae (Lepidoptera) from a Montane Rainforest in Southern Ecuador

**DOI:** 10.1673/031.010.6701

**Published:** 2010-06-15

**Authors:** Florian Bodner, Gunnar Brehm, Jürgen Homeier, Patrick Strutzenberger, Konrad Fiedler

**Affiliations:** ^1^ Department of Animal Biodiversity, Rennweg 14, University of Vienna, 1030 Vienna, Austria; ^2^ Institut für Spezielle Zoologie und Evolutionsbiologie mit Phyletischem Museum, Erbertstraße 1, Friedrich-Schiller- Universität Jena, 07743 Jena, Germany; ^3^ Plant Ecology, Untere Karspüle 2, Georg-August University of Göttingen, 37073 Göttingen, Germany

**Keywords:** Andes, host plant affiliations, insect herbivores, larval morphology, pupal morphology, Neotropical Caterpillars

## Abstract

During four months of field surveys at the Reserva Biológica San Francisco in the south Ecuadorian Andes, caterpillars of 59 Geometridae species were collected in a montane rainforest between 1800 and 2800m altitude and reared to adults. The resulting data on host plant affiliations of these species was collated. The preimaginal stages of 58 and adult stages of all 59 species are depicted in colour plates. Observations on morphology and behaviour are briefly described. Five species, documented for the first time in the study area by means of larval collections, had not been previously collected by intensive light-trap surveys. Together with published literature records, life-history data covers 8.6% of the 1271 geometrid species observed so far in the study area. For 50 species these are the first records of their early stages, and for another 7 the data significantly extend known host plant ranges. Most larvae were collected on shrubs or trees, but more unusual host plant affiliations, such as ferns (6 geometrid species) and lichens (3 geometrid species), were also recorded. Thirty-four percent of the caterpillars were infested by wasp or tachinid parasitoids.

## Introduction

Herbivorous insects are important primary consumers of plant biomass in terrestrial ecosystems. They also comprise a major fraction of animal biodiversity on earth (Meyhew 2001; Novotny et al. 2004). The diversity of tropical herbivorous arthropods has been at the centre of an ongoing debate about the magnitude of global animal species richness. While available estimates agree that true species richness is far higher than the number of species currently described, there is considerable controversy regarding the extent to which the various estimates diverge. Levels of host plant specificity in herbivorous insects play a critical role in all these estimates (Erwin 1982; Ødegaard 2000; Novotny et al. 2002). It is still debated whether host specificity is higher in tropical rather than temperate ecosystems (e.g. Fiedler 1998; Novotny and Basset 2005; Novotny et al. 2006; as opposed to: Dyer et al. 2007). However, what most authors tend to agree on is a severe lack of data, hindering a thorough investigation of this topic. Consequently the gathering of additional host plant data can be seen as the obvious step for improving our understanding of biodiversity, ecology and evolution of tropical herbivorous insects (Scriber 2002; Novotny and Basset 2005). This study aims to increase available host plant data for Neotropical representatives of the lepidopteran family Geometridae, at an Andean site where large-scale ecosystem studies are currently under way (Beck et al. 2008a).

## Material and Methods

### Study organisms

Geometridae are one of the three largest families of Lepidoptera, with a global total of more than 21000 described species, 6450 of which occur in South America (Scoble 1999). Montane forests in the Ecuadorian Andes were recently identified as one of the global hotspots for geometrid species diversity (Brehm et al. 2005), with 1266 recorded species (and an estimated total of >1450 species) occurring in one relatively small nature reserve (Brehm et al. 2005, see below). Brehm (2002) collated host plant records for 48 geometrid species that occur in the study area, gathered mainly from his own caterpillar collections (Brehm 2003) and supplemented by web-based sources (Janzen and Hallwachs 2009, Robinson et al. 2009). Since then, host plant records for 11 more species occurring in the study area have been reported (Dyer and Gentry 2009; Dyer et al. 2009), increasing the data set to 59 species (=4.7% of the local species list).

### Study area and field work

Data were collected in the Reserva Biológica San Francisco (RBSF), a privately owned nature reserve adjacent to Podocarpus National Park (since 2007 part of the UNESCO biosphere reserve “Podocarpus-El Condor”) in southern Ecuador (province Zamora-Chinchipe). The study area, located on the eastern slope of the Andes, has been the target of intensive ecological research since 1997 (Beck et al. 2008a). Caterpillar samples were taken at an elevation ranging from 1800–2800m above sea level. However, the majority of the data was collected at an elevation of 1800–2100m, in close proximity to the Estación Científica San Francisco (3°58′ S, 79°05′ W), during the period from February 1 to May 28, 2006.

RBSF is covered by nearly pristine montane rain forest (Beck et al. 2008b; Homeier et al. 2008). Its moth fauna has been studied intensively (1999–2006) by light-trapping, offering insight into patterns of moth diversity and community structure (e.g.: Brehm et al. 2003; Fiedler et. al. 2008; Hilt and Fiedler 2008).

Caterpillars were collected by visually scanning vegetation during the day and by searching with lights during the night, as well as by common plant-beating techniques (e.g. Schauff 1986; Leather 2005). Caterpillars were transferred to the laboratory and kept in plastic boxes. These were lined with damp paper towel to maintain high humidity. Caterpillars were fed with their appropriate host plant until pupation. Old food plant material was replaced every 2–3 days. Pupae were kept in similar plastic boxes until adult emergence.

One species, *Pantherodes conglomerata*, was reared from eggs obtained from a light-caught adult female. In this case, several plants were experimentally offered to find a suitable host plant.

### Documentation

Larval stages were documented using digital photographs, taken from different aspects on a regular basis and whenever developmental changes were observed. For size measurements, scaled paper was used as background for caterpillar photographs. Host plants were also photographed. Pictures were taken with a Canon EOS 300D digital single lens reflex camera (3072 × 2048 pixel resolution), equipped with a Sigma 105mm F2.8 EX DG macro lens and a Sigma EM-140 DG macro flash.

### Data processing and identification

Reared moths were identified to species when possible, or were sorted as morphospecies by comparison with previously identified specimens or with digital photographs of identified material. Caterpillars that failed to develop into adults were preserved in 70% ethanol, as were pupal exuviae and parasitoids that emerged in captivity. Nomenclature follows Pitkin (2002, 2005) for members of the subfamily Ennominae and Scoble (1999) for all remaining species. Morphospecies ID codes of Brehm et al. (2005) were used. New codes were assigned to five species previously unknown from the study area.

Hymenopteran parasitoids were identified to family level using the keys to the Hymenoptera of Costa Rica (Hanson and Gauld, 1995). One specimen was identified to family by Martin Schwarz (Biologiezentrum Linz-Dornach, Austria).

Host plants were identified from pictures taken in the field, in some cases with the help of the online data base Visual Plants (Homeier and Dalitz 2009). Plant family delimitations follow APG II (2003). Moth vouchers have been deposited in the research collection of G. Brehm at the Phyletisches Museum of the Zoological Institute, University of Jena, Germany.

## Results and Discussion

### Overview and rearing success

During the field surveys 325 caterpillars of Geometridae were collected. Of these caterpillars, 105 individuals were successfully reared to adults. Four specimens of two *Eois* species (Larentiinae) that failed to develop were assigned to morphospecies, known from previous light trapping, by means of DNA barcoding. For barcoding we used a 676 bp fragment in the 5' part of the mitochondrial cytochrome oxidase subunit I (COI) gene (Valentini et al. 2008). Neighbour-joining clustering provided unambiguous assignments of the larval sequences to sequences from adult samples gathered by Strutzenberger (2010). Altogether, the surveys resulted in information on host plant associations and caterpillar morphology for 59 geometrid species (43 Ennominae, 2 Sterrhinae, and 15 Larentiinae). A search of the literature, as well as web-based databases, suggests that for 50 of these species, these are the first reports available.

Two hundred and twenty caterpillars (68%) died in captivity: 54 (17%) due to the emergence of parasitoids, 166 (51%) from infections, moulting difficulties, handling mistakes, or food refusal. The overall rearing success (32%) was surprisingly close to the 33% success rate reported by Brehm (2003). Of the 220 caterpillars that failed to develop into adults, 81 (37%) could be reliably identified through caterpillar morphology, and four individuals representing 3 species were identified through DNA barcoding. The remaining 139 caterpillars represented approximately 40–50 additional morphos-pecies.

### Parasitoids

Of the 325 caterpillars collected, 54 (17%) produced larvae or imagines of parasitoid wasps and flies. The parasitoids included 31 solitary hymenopterans (22 Braconidae, 8 Ichneumonidae, and 1 Chalcididae). One caterpillar produced three hymenopterans of the family Eulophidae. Seven hymenopteran individuals from five caterpillars failed to develop to adulthood and could not yet be assigned to family.

In addition, 11 representatives of the family Tachinidae successfully developed to the adult stage, one failed to develop. Another five solitary parasitoids, that did not develop successfully, could not be reliably assigned to either Hymenoptera or Diptera. No incidences of multiparasitism or superparasitism were observed (see Godfray 1994). If caterpillars that died from unknown reasons or by accident are excluded, the overall incidence of parasitism increased to 54 of 159 individuals (= 34%). However, due to the small sample size and the unstandardized sampling approach, these figures can only be viewed as a very crude gross estimate for parasitation rates of the whole family of Geometridae in this area.

### Host plants

In general, all plants within the lower 2–3 m of vegetation were searched for caterpillars, but some plant taxa received particular attention. These included the genus *Piper* (Piperaceae) for investigation of the putative association between these plants and the geometrid genus *Eois* (see Dyer and Palmer 2004), the family Asteraceae, and the fern genus *Pteridium* (Dennstaedtiaceae). The latter are dominant plant taxa in disturbed habitats of the study area and are therefore of high ecological interest for understanding forest regeneration in the Andes of southern Ecuador (Beck et al. 2008c; Hartig and Beck 2003).

Table 1 presents the resulting data on host plant affiliation, together with information on the approximate body length of the last instar caterpillars and references to additional records on the preimaginal stages of each species (if available).

Our observations increase the number of Geometridae species from the Reserva Biológica San Francisco, for which life-history information is available, from 59 to 109. This amounts to 8.6% of the 1271 species recorded so far in the general study area, and to 10.2% (100 species) if the fauna is restricted to the 977 species so far recorded at the elevation covered by this research.

**Table 1.**
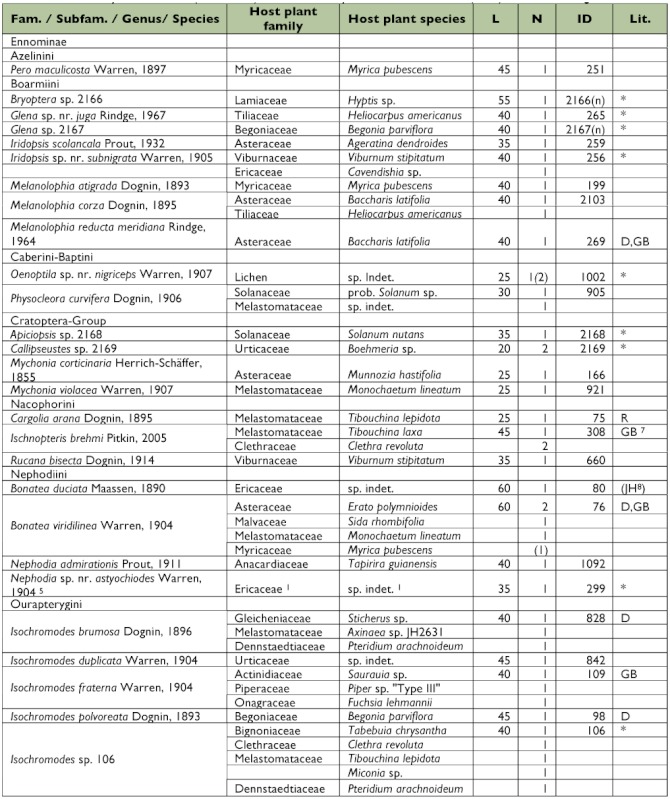
Reared Geometridae species, sorted by subfamily, tribe and genus. For every species the number of emerged adults and the number of assigned dead caterpillars (in brackets) are shown. Also information on host plants and approximate body length in mm (L) of the last instar larvae before entering the prepupal phase is listed. ID numbers follow BREHM et al. (2005, electronic supplement). In five cases, new IDs were assigned to species not previously recorded in the study area; all five species are so far identified at generic level. References to available literature on the caterpillars or host plant affiliations are coded: GB: BREHM (2003), JH: JANZEN & HALLWACHS (2008), D: DYER et al. (2008) and R: ROBINSON et al. (2008). Species that could not yet be formally identified and therefore could not be compared with literature are marked with *. Geometrid taxonomy follows Pitkin (2002, 2005) for the subfamily Ennominae and Scoble (1999) for all remaining subfamilies.

**Table:**
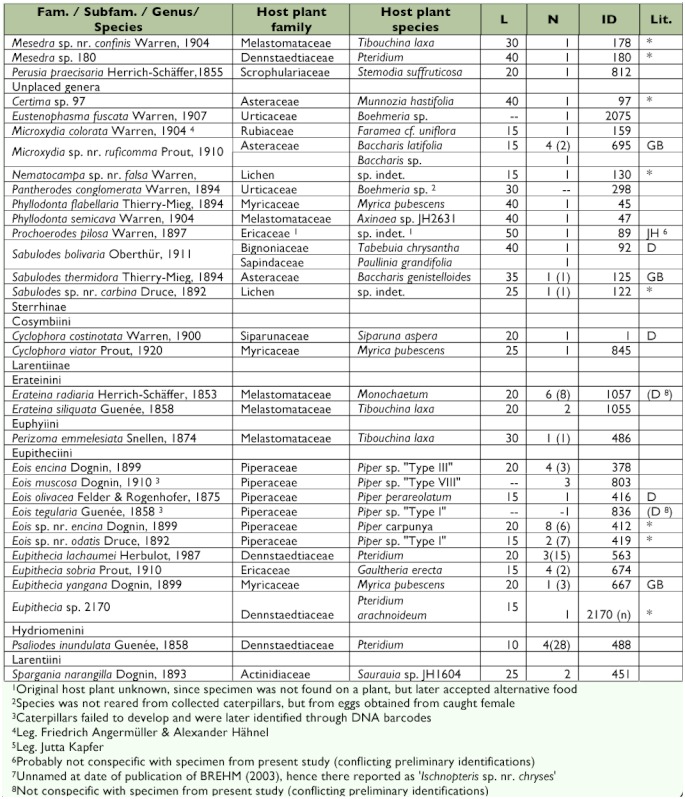

### Remarks on host plant affiliations

Two thirds of the moth species covered in this study were encountered only once, therefore those host plant affiliations must be interpreted with care. It may sometimes occur that a species is found and successfully reared on a plant, which is not part of its natural host plant range. On the other hand, even single records may provide important insight into patterns of host use if they can be placed in a comparative context.

In our study, a high number of species use host plants or food sources that are considered unusual for Geometridae (and for macromoths in general), such as ferns (e.g. Hendrix 1980; Lawton 1982; Weintraub et al. 1995) and lichens (e.g. Lawrey 1987; Hesbacher et al. 1995). Of the 59 species treated in this study, six (10%) were recorded from bracken fern (*Mesedra* sp. [180, see Table 1], *Eupithecia lachaumei*, *Eupithecia* sp. [2170], *Psaliodes inundulata*, *Isochromodes brumosa*, *Isochromodes* sp. [106]). *Isochromodes brumosa* feeds on umbrella fern (genus *Sticherus*, Gleicheniaceae) in addition to bracken fern.

Fern feeding in the Geometridae is confined to a few isolated specialists or to small subclades (e.g. Ennominae-Lithinini: Holloway 1987; Weintraub et al. 1995), although polyphagous feeders of spermatophytes occasionally accept ferns as minor hosts (Hendrix 1980; Robinson et al. 2009). While Hendrix (1980) described ferns as generally underutilized, Lawton (1982) pointed out that bracken fern does support a sizable fauna of herbivores, at least in parts of its distribution. Three geometrid species (5%) were recorded on lichens (*Sabulodes* sp. nr. *carbina*, *Perusia* sp. nr. *nigriceps*, *Nematocampa* sp. nr. *falsa*).

The species-rich genus *Eois* (Geometridae: Larentiinae) is of particular interest for questions regarding host plant specialization. *Eois* species are hypothesized to specialize on *Piper* (Piperacee) throughout the Neotropics (e.g. Dyer and Palmer 2004). In our study, caterpillars of four *Eois* species were reared to adults and caterpillars of two more were identified by DNA barcodes. All were found exclusively on *Piper*. Each *Eois* was encountered only on a single species of *Piper*, and only *E*. sp. nr. *odatis* and *E. tegularia* shared the same host plant.

*Eois olivacea* feeds on two different hostplants in northeastern Ecuador (Dyer et al. 2009): *Piper baezanum*, which appears very similar to the host plant recorded in our study and *Piper longifolium*. However, different caterpillar color morphs are consistent in feeding each *Piper*, suggesting that *‘Eois olivacea'* might comprise more than one species (G. Rodríguez and L. Dyer, personal communication). This is supported by DNA barcoding results and phylogenetic analysis using a combination of nuclear and mitochondrial genes (Strutzenberger 2010) as well as by caterpillar records from our study site (F. Bodner, unpublished data).

### Observations on morphology and Behavior

**Figure 1.**
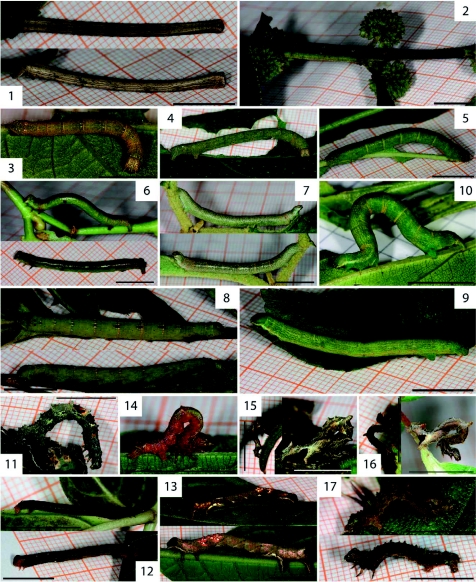
*Pero maculicosta* Warren, 1897. **Figure 2.** *Bryoptera* sp. [2166]. **Figure3.** *Glena* sp. nr. *juga* Rindge, 1967. **Figure 4.** Glena sp. [2167]. **Figure 5.** *Iridopsis scolancala* Prout, 1932. **Figure 6–7.** *Iridopsis* sp. nr. *subnigrata* Warren, 1905. **Figure 8.** *Melanolophia atigrada* Dognin, 1893. **Figure 9.** *Melanolophia corza* Dognin, 1895. **Figure 10.** *Melanolophia reducta meridiana* Rindge, 1964. **Figure 11.** *Oenoptila* sp. nr. *nigriceps* Warren, 1907. **Figure 12.** *Physocleora curvifera* Dognin, 1906. **Figure 13.** *Apiciopsis* sp. [2168]; probably L4 (top) and L5 (bottom). **Figure 14.** *Callipseustes* sp. [2169]; probably L5. **Figure 15.** *Mychonia corticinaria* Herrich-Schäffer, 1855; probably L4 (left) and L5 (right). **Figure 16.** *Mychonia violacea* Warren, 1907; probably L4 (left) and L5 (right). **Figure 17.** *Cargolia arana* Dognin, 1895. Caterpillars are last instars except where indicated differently. The scale bars are 1 cm in length, except where indicated as “1/2”. High quality figures are available online.

In general, geometrid caterpillars are cryptic in appearance, with brown, green and grey tones predominating. Some resemble twigs (e.g.: *Pero maculicosta*, Figure 1), lichens (e.g.: *Oenoptila* sp. cf. *nigriceps*, Figure 11; *Nematocampa* sp. nr. *falsa*, Figure 39), or mossy bark (e.g. *Phyllodonta semicava*, Figure 42; *Cargolia arana*, Figure 17), while others seem to be inconspicuously coloured to the human eye. Whether their crypsis pertains to particular predators, such as visually hunting birds, requires further assessment (Church et al. 1998). An account of notable observations on certain species is given below.

The two specimens of *Iridopsis* sp. nr. *subnigrata* differ strongly in colouration (Figure 6–7) remarkably matching that of their respective host plants. *Callipseustes* sp. [2169] (Figure 14) shows bright red colouration making it conspicuous against the green leaves of its host plant (*Boehmeria* species, family Urticaceae), but matching the red twigs.

The caterpillars of *Mychonia violacea* (Figure 16) and *Mychonia corticinaria* (Figure 15) show similar colouration, but differ in the number and shape of their dorsal protuberances. However, adult wing pattern and colouration in both specimens (Figure 87 and Figure 88) is similar. Further morphological studies are required in this species complex.

**Figure 18.**
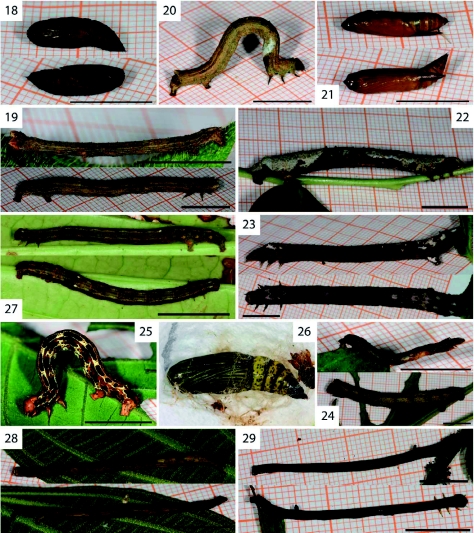
*Cargolia arana* Dognin, 1895; pupa. **Figure 19.** *Ischnopteris brehmi* Pitkin, 2005; probably L4 (top) and L5 (bottom). **Figure 20–21.** *Rucana bisecta* Dognin, 1914; L5 (Figure 20) and pupa (Figure 21). **Figure 22.** *Bonatea duciata* Maassen, 1890. **Figure 23–24.** *Bonatea viridilinea* Warren, 1904; L5 (Figure 23 and 24 bottom); L3 or L4 (Figure 24 top). **Figure 25–26.** *Nephodia admirationis* Prout, 1911; L5 (Figure 25) and pupa (Figure 26). **Figure 27.** *Nephodia* sp. nr. *astyochiodes* Warren, 1904. **Figure 28.** *Isochromodes brumosa* Dognin, 1896. **Figure 29.** *Isochromodes duplicata* Warren, 1904. High quality figures are available online.

Caterpillars of *Ischnopteris brehmi* (Figure 19) have lateral bulges at at the 2^nd^ abdominal segment. Pupae of *I. brehmi* (pupae not shown) and *Rucana bisecta* (Figure 21) have the antenna and trunk sheaths extended to a spike that reaches down to about half of the free moving portion of the abdomen. In the third reared Nacophorini, *Cargolia arana* (Figure 18), the antenna and trunk sheaths extended only beyond the wing sheaths. This feature was not mentioned by Rindge (1983) in his revision of the Nacophorini or by Pitkin (2002, 2005), but may be a synapomorphy for the tribe. On the other hand, Parra and Henriquez-Rodriguez (1993) showed the pupae of two Nacophorini (*Mallomus falcatus* and *M. tumidus*) that lacked this feature, possibly suggesting that this character state is restricted to a smaller clade within the Nacophorini.

**Figure 30.**
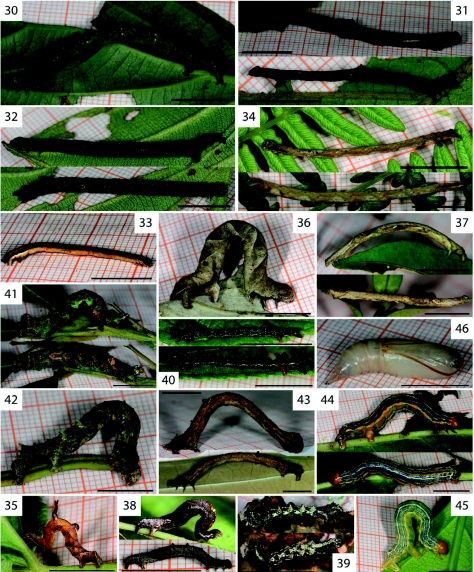
*Isochromodes fraterna* Warren, 1904. **Figure 31.** *Isochromodes polvoreata* Dognin, 1893. **Figure 32.** *Isochromodes* sp. [106]. **Figure 33.** *Mesedra* sp. nr. *confinis* Warren, 1904.**Figure 34.** *Mesedra* sp. [180]. **Figure 35.** *Perusia praecisaria* Herrich-Schäffer,1855. **Figure 36.** *Certima* sp. [97]. **Figure 37.** *Microxydia colorata* Warren, 1904. **Figure 38.** *Microxydia* sp. nr*. ruficomma* Prout, 1910. **Figure 39.** *Nematocampa* sp. nr. *falsa* Warren, 1906. **Figure 40.** *Pantherodes conglomerata* Warren, 1894; probably L4 (top) and L5 (bottom). **Figure 41.** *Phyllodonta flabellaria* Thierry-Mieg, 1894. **Figure 42.** *Phyllodonta semicava* Warren, 1904. **Figure 43.** *Prochoerodes pilosa* Warren, 1897. **Figure 44–46.** *Sabulodes bolivaria* Oberthür, 1911; L5 (Figure 44–45) and pupa (Figure 46). High quality figures are available online.

Five species that, according to their adult morphology, belong in the genus *Isochromodes*, showed divergent caterpillar morphology. Caterpillars of *I. duplicata* (Figure 29) have two black bulges on the 5^th^ abdominal segment that were present in all instars. The caterpillar of *I. polvoreata* (Figure 31) has the third thoracic leg elevated on an enlarged basis and a dorsal and lateral protuberance on the ^rd^ abdominal segment. Together these made the caterpillar resemble a dead twig with two leaf scars. Caterpillars of *Isochromodes* sp. [106] (Figure 32) and *I. fraterna* (Figure 30) varied in a similar range of green and brown colours with or without white and brown dorsal markings. No clear diagnostic characters could be identified.

The caterpillar of *Perusia praecisaria* (Figure 35) shows two thin elongated dorsal protuberances: a long one forking into two points, and a shorter one on the 2^nd^ and 3^rd^ abdominal segments, respectively. The caterpillar is similar to that of *Melinodes subapicata* (see Brehm 2003). *Perusia* and *Melinodes* are currently placed in different tribes (Ourapterygini and *Cratoptera*-group, respectively, Pitkin 2002). The striking similarity between *Perusia praecisaria* and *Melinodes subapicata* caterpillars instead suggests a close relationship between these species. Further studies in both genera as well as in related taxa are required to resolve the relationships between the Ourapterygini and the *Cratoptera*-group.

The *Microxydia colorata* caterpillar was of pronouncedly different appearance than those of *Microxydia* sp. nr. *Ruficomma*. On adult morphology, Pitkin (2002) stated that “*M. colorata* has a more complex wing pattern unlike that of other *Microxydia* species.” Taken the differences of adult and larval morphology into accout, we suggest that *M. colorata* is misplaced within this genus. Further morphological studies are required.

**Figure 47.**
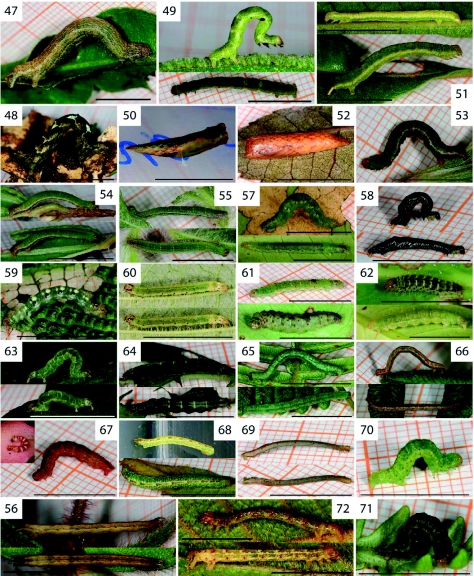
*Sabulodes thermidora* Thierry-Mieg, 1894. **Figure 48.** *Sabulodes* sp. nr. *carbina* Druce, 1892; probably F5. **Figure 49–50.** *Cyclophora costinotata* Warren, 1900; L5 (Figure 49) and pupa (Figure 50). **Figure 51–52.** *Cyclophora viator* Prout, 1920; L5 (Figure 51) and pupa (Figure 52). **Figure 53–54.** *Erateina radiaria* Herrich-Schäffer, 1853. **Figure 55.** *Erateina siliquata* Guenée, 1858. **Figure 56.** *Perizoma emmelesiata* Snellen, 1874. **Figure 57.** *Eois encina* Dognin, 1899. **Figure 58.** *Eois muscosa* Dognin, 1910; probably L4 or L5. **Figure 59.** *Eois olivacea* Felder and Rogenhofer, 1875. **Figure 60 & 63.** *Eois tegularia* Guenée, 1858; probably L4 or L5. **Figure 61–62.** *Eois* sp. nr. *encina* Dognin, 1899. **Figure 64.** *Eois* sp. nr. *odatis* Druce, 1892. **Figure 65–66.** *Eupithecia lachaumei* Herbulot, 1987; probably L5 (Figure 65) and L4 or L5 (Figure 66). **Figure 67.** *Eupithecia sobria* Prout, 1910; L2 or L3 (left) and L5 (right). **Figure 68.** *Eupithecia yangana* Dognin, 1899; L3 or L4 (top) and L4 or L5 (bottom). **Figure 69.** *Eupithecia* sp. [2170]; probably L4. **Figure 70–71.** *Psaliodes inundulata* Guenée, 1858. **Figure 72.** *Spargania narangilla* Dognin, 1893. High quality figures are available online.

**Figure 73.**
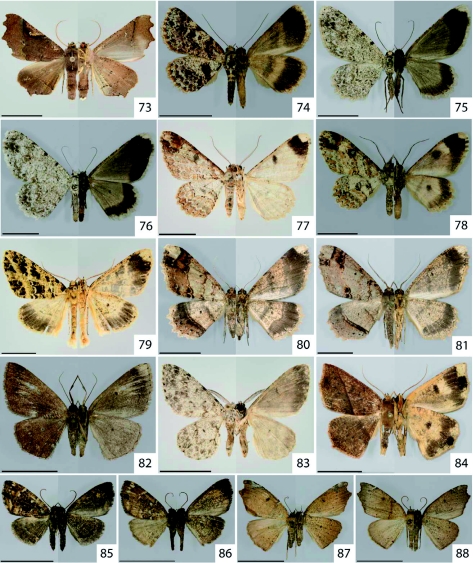
*Pero maculicosta* Warren, 1897. **Figure 74.** *Bryoptera* sp. [2166]. **Figure 75.** *Glena* sp. nr. *juga* Rindge, 1967. **Figure 76.** Glena sp. [2167]. **Figure 77.** *Iridopsis scolancala* Prout, 1932. **Figure 78.** *Iridopsis* sp. nr. *subnigrata* Warren, 1905. **Figure 79.** *Melanolophia atigrada* Dognin, 1893. **Figure 80.** *Melanolophia corza* Dognin, 1895. **Figure 81.** *Melanolophia reducta meridiana* Rindge, 1964. **Figure 82.** *Oenoptila* sp. nr. *nigriceps* Warren, 1907. **Figure 83.** *Physocleora curvifera* Dognin, 1906. **Figure 84.** *Apiciopsis* sp. [2168]. **Figure 85–86.** *Callipseustes* sp. [2169]. **Figure 87.** *Mychonia corticinaria* Herrich-Schäffer, 1855. **Figure 88.** *Mychonia violacea* Warren, 1907. Pictures show both dorsal (left half) and ventral (right half) side of same individual. High quality figures are available online.

**Figure 89.**
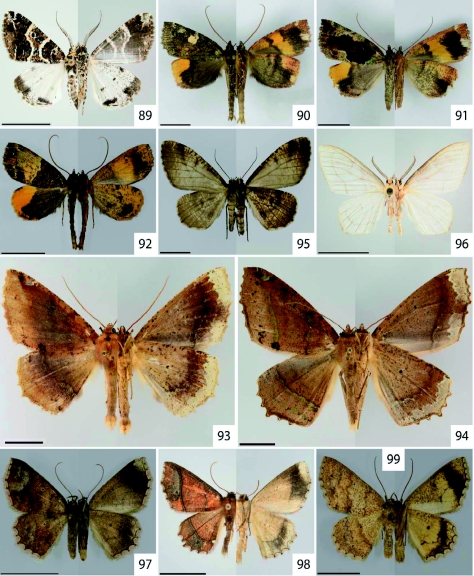
*Cargolia arana* Dognin, 1895. **Figure 90–91.** *Ischnopteris brehmi* Pitkin, 2005. **Figure 92.** *Rucana bisecta* Dognin, 1914. **Figure 93.** *Bonatea duciata* Maassen, 1890. **Figure 94.** *Bonatea viridilinea* Warren, 1904. **Figure 95.** *Nephodia admirationis* Prout, 1911. **Figure 96.** *Nephodia* sp. nr. *astyochiodes* Warren, 1904. **Figure 97.** *Isochromodes brumosa* Dognin, 1896. **Figure 98.** *Isochromodes duplicata* Warren, 1904. **Figure 99.** *Isochromodes fraterna* Warren, 1904. High quality figures are available online.

**Figure 100.**
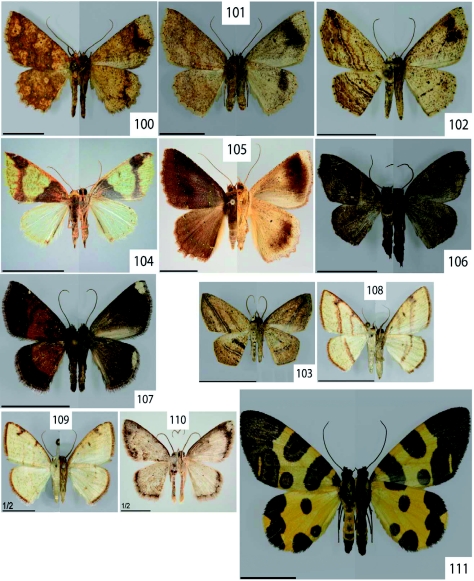
*Isochromodes polvoreata* Dognin, 1893. **Figure 101.** *Isochromodes* sp. [106]. **Figure 102.** *Mesedra* sp. nr. *confinis* Warren, 1904.**Figure 103.** *Mesedra* sp. [180]. **Figure 104.** *Perusia praecisaria* Herrich-Schäffer,1855. **Figure 105.** *Certima* sp. [97]. **Figure 106.** *Eustenophasma fuscata* Warren, 1907. **Figure 107.** *Microxydia colorata* Warren, 1904. **Figure 108–109.** *Microxydia* sp. nr. *ruficomma* Prout, 1910. **Figure 110.** *Nematocampa* sp. nr. *falsa* Warren, 1906. **Figure 111.** *Pantherodes conglomerata* Warren, 1894. High quality figures are available online.

**Figure 112.**
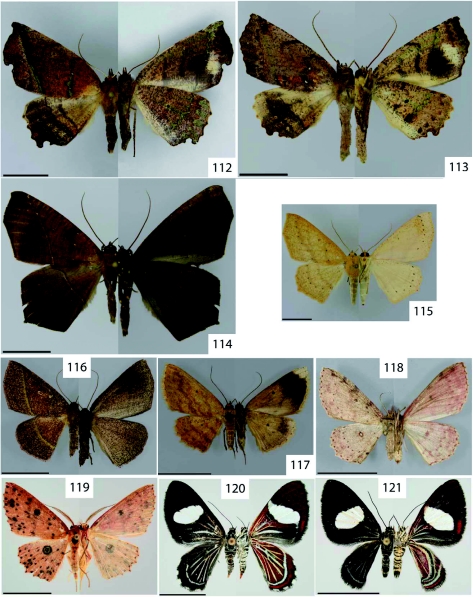
*Phyllodonta flabellaria* Thierry-Mieg, 1894. **Figure 113.** *Phyllodonta semicava* Warren, 1904. **Figure 114.** *Prochoerodes pilosa* Warren, 1897. **Figure 115.** *Sabulodes bolivaria* Oberthür, 1911. **Figure 116.** *Sabulodes thermidora* Thierry- Mieg, 1894. **Figure 117.** *Sabulodes* sp. nr. *carbina* Druce, 1892. **Figure 118.** *Cyclophora costinotata* Warren, 1900. **Figure 119.** *Cyclophora viator* Prout, 1920. **Figure 120.** *Erateina radiaria* Herrich-Schäffer, 1853. **Figure 121.** *Erateina siliquata* Guenée, 1858. High quality figures are available online.

**Figure 122.**
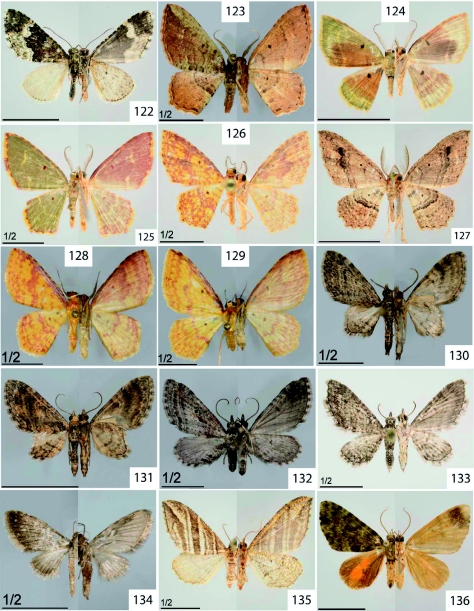
*Perizoma emmelesiata* Snellen, 1874. **Figure 123.** *Eois encina* Dognin, 1899. **Figure 124.** *Eois muscosa* Dognin, 1910. **Figure 125.** *Eois olivacea* Felder and Rogenhofer, 1875. **Figure 126 & 128.** *Eois tegularia* Guenée, 1858. **Figure 127.** *Eois* sp. nr. *encina* Dognin, 1899. **Figure 129.** *Eois* sp. nr. *odatis* Druce, 1892. **Figure 130–131.** *Eupithecia lachaumei* Herbulot, 1987. **Figure 132.** *Eupithecia sobria* Prout, 1910. **Figure 133.** *Eupithecia yangana* Dognin, 1899. **Figure 134.** *Eupithecia* sp. [2170]. **Figure 135.** *Psaliodes inundulata* Guenée, 1858. **Figure 136.** *Spargania narangilla* Dognin, 1893. High quality figures are available online.

The caterpillar of *Prochoerodes pilosa* (Figure 43) displayed a behaviour of pulling the first three segments together to form a uniform knob with the head and thoracic legs (Figure 43 top). The caterpillar thus mimicked a dead twig or leaf stalk. *Sabulodes bolivaria* (Figure 44–46) was found in two individuals. One (Figure 44), found as an early instar, developed a rather intensive, dark colouration. The other caterpillar (Figure 45), found in its last instar, had a pale colouration with some parts of the pattern completely missing. Both caterpillars preferred to hide within a leaf-tent, as mentioned by Brehm (2003) for this genus.

Two *Cyclophora* species (*C. costinotata*, Figure 49–50 and *C. viator*, Figure 51–52) were reared. In both, the anteriorly truncated pupae bear a silk girdle around the 3^rd^ abdominal segment (Figure 50 and 52). This trait is supposedly apomorphic for the genus *Cyclophora* and some, possibly all, other genera in the tribe Cosymbiini (Holloway 1997; Sihvonen and Kaila 2004).

Most *Eois* caterpillars show some shade of green and have black, sometimes also white markings (Figure 57–64). The amount of these markings generally increased with development, but was also variable between individuals within the species *Eois encina* (Figure 57) and *Eois* sp. nr. *encina* (Figure 61–62). Images of several *Eois* species shown by Dyer and Gentry (2009), Dyer at al. (2009) and Janzen and Hallwachs (2009) provide further examples of pronounced intraspecific variation.

The status of *Eupithecia lachaumei* is uncertain, where pronounced variation in caterpillar coloration (Figure 65–66) is paralleled by subtle differences in wing pattern (Figure 130–131), possibly pointing to the existence of a species complex. Most caterpillars of *Psaliodes inundulata* are green (Figure 70), but a few showed diffuse dark patterns making them almost black (Figure 71).

*Spargania narangilla* (Figure 72) displayed interesting pupation behaviour; caterpillars build their cocoon in a hole in the leaf and covered it with chewed bits of leaf (data not shown). This gave the impression of a dead leaf area, rather than a cocoon.

In general, the geometrid larvae we studied are ectophagous, except that caterpillars of *Eupithecia sobria* feed inside the flowers of their host plant in the early stages (Figure 67 left). Later instars (Figure 67 right) feed partially exposed on the flowers and fruits.

## Conclusions

With these results, the number of Geometridae species from the Reserva Biológica San Francisco, for which life-history information is now available, nearly doubled. The simple sampling methods used in this study have the potential to unravel many novel facets of the interactions between herbivorous insects and their host plants in Andean rain forests. These forests suffer heavily from ongoing deforestation (Beck et al. 2008a; Mosandl et al. 2008). Therefore, the Andean fauna deserves more intense research so that, as additional information is gathered, this will allow for more in-depth comparisons with ongoing studies at the well established focal sites in tropical lowland forests (e.g., Janzen and Hallwachs 2009; Novotny et al. 2009).
